# *Enterobius vermicularis* in the Endometrium of the Uterus: A Case Report

**Published:** 2017

**Authors:** Biserka PIGAC, Silvija MAŠIĆ, Valentina MAŠIĆ

**Affiliations:** 1. Pathology, Cytology and Forensic Medicine Unit, Varaždin General Hospital, Varaždin, Croatia; 2. Clinic for Oncology and Nuclear Medicine, University Hospital Center Sestre Milosrdnice, Zagreb, Croatia; 3. Training and Education Center ‘Vinko Bek’, Zagreb, Croatia

**Keywords:** *Enterobius vermicularis*, Ectopic parasite, Endometrium, Uterus, Croatia

## Abstract

*Enterobius vermicularis* is an intestinal nematode of humans and the most common helminth infection. Main transmission path is direct contact between infected and uninfected person meaning ingestion of the eggs. Human infections are usually asymptomatic or manifest as perianal itching. Although ectopic locations are uncommon, *Enterobius* can occasionally be detected in appendix, kidney, male urinary tract and female genital tract. We present a case from Varaždin General Hospital, Varaždin, Croatia in 2012, involving a 90-yr-old female patient who underwent hysterectomy leading to accidental finding of *E. vermicularis* in the uterus despite being asymptomatic for enterobiasis. Since there were no signs and symptoms of parasitic infection, no antiparasitic drugs were administered. Parasite was not observed during macroscopic examination, yet microscopic examination of the material demonstrated helminth within endometrium surrounded by dense inflammatory infiltrate, predominantly lymphocytes and some eosinophils. Internal structures of the parasite were collapsed, while well-developed musculature and cuticle were preserved. We present this case to educate and remind physicians on this parasitosis as possible diagnosis. Although non-gastrointestinal locations of *Enterobius* infestation are rare, this infection should be considered in patients with abdominal pain, genitourinary symptoms, and pelvic pain in order to apply appropriate treatment and prevent further complications.

## Introduction

Enterobiasis is common, highly contagious helminthic infection of gastrointestinal tract (1–3) spread mostly in Western Europe and North America (4) which usually affects children (3). It usually presents as asymptomatic condition and is often considered only in cases of traveling to endemic areas which results in lower degree of recognition of this entity as differential diagnosis (1). Ectopic localizations of *Enterobius* are uncommon and usually appear as incidental finding in tissue samples of asymptomatic patients (5). Some of those reported are peritoneal cavity, female genital tract, lung, liver, prostate, renal pelvis (5). Correct prevalence in female genital tract is not familiar (6).

We present a case of accidental finding of *E. vermicularis* in the endometrium of the uterus in a patient without data on traveling to this pinworm endemic areas.

## Case report

A 90-yr-old female patient underwent elective hysterectomy due to descensus uteri in Varaždin General Hospital, Varaždin, Croatia, in 2012. She complained only about heaviness feeling in pelvic area. She had no previous surgeries or illnesses, took no medication and reported no allergies. The patient recovered well after surgical procedure and was discharged from hospital. Informed consent was obtained from the patient. No personal data which would in any way violate the patient’s privacy or reveal patient’s identity, were used in the study.

**Fig.1:**
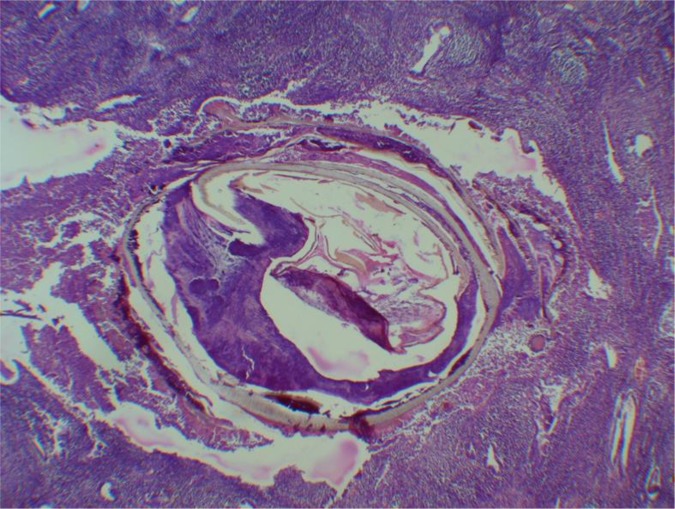
*Enterobius vermicularis* in the endometrium of the uterus surrounded by inflammatory cells (H&E, 10×)

We received removed uterus which was 6 cm long, 4 cm wide and 3 cm in anteroposterior diameter. Thickness of the endometrium was 0.4 cm. Macroscopic examination revealed small, atrophic uterus with no conspicuous pathological lesions. A sample of the tissue was routinely taken for further pathohistological analysis. H&E sections revealed presence of helminth within endometrium surrounded by dense inflammatory infiltrate, mostly lymphocytes with some eosinophils (Fig.1).

We identified the parasite based on its morphology on H&E sections. Well-developed musculature and cuticle of the parasite were preserved, yet internal structures were not kept. According to patient’s medical history, she complained of no medical problems that would indicate pinworm infestation. However, no data on traveling to this helminths-endemic areas were obtained. The patient was asymptomatic for enterobiasis and that parasite finding was accidental, there was no need for use of antihelminthics. During 6 months follow-up no signs of helminth infestation were observed.

## Discussion

*E. vermicularis*, also referred to as pinworm, oxyuris or threadworm (7) is helminth characterized by low pathogenicity and therefore most of infections are asymptomatic (4, 5).

*Enterobius* is transmitted by ingestion of ova through contaminated hands or food which leads to egg disolvement and release of larvae. Then fertilization of mature female worms occurs (usually in caecum or terminal ileum) who then migrate to perianal region and lay eggs resulting in itching. Scratching of the infested area leads to further contamination of hands and transmission of the parasite to others and the host (4, 8). Larvae from the eggs can also migrate from the anus to gastrointestinal tract where they mature (8). Another possible way of infection is inhalation and swallowing of airborne eggs (7).

Adults of *Enterobius* come to female genital tract from perineum to vagina and therefore to other parts of genital tract (5). Infestation of female genital tract is unusual (7) and most often presents as accidental finding of ova on cervical smears or as vulvovaginitis, salpingitis, pelvic pain, pelvic masses, irregular menstrual cycles, postmenopausal bleeding (2, 4, 5).

Other extraintestinal manifestations of enterobiasis are also rare. Some of them are appendicitis, recurrent urinary infections, peritonitis, abdominal pain (7, 9, 10).

In some cases, formation of granuloma in the uterus can occur and should be taken into consideration since it resembles malignant lesions, fibroma, leiomyoma, endometrioma (5). However, eggs of *Enterobius* can be confused with *Schistosoma hematobium*, fungal spores, *Entamoeba histolytica*, *Microfilaria*, *Strongyloides stercoralis*, *Trichuris trichiura*, *Ascaris lumbricoides* and *Taenia* eggs (6, 7).

Diagnosis of *E. vermicularis* infestation is established by performing cello-tape anal swab technique in order to identify *Enterobius* eggs on microscopic examination (3). Eggs are characterized by orange-red staining with Papanicolaou stain and flattened on one side with dimensions about 25×50 microns (8, 11). In case of ectopic localizations diagnosis can only be established by histopathologic analysis since lesions presenting as granulomas with parasite ova, inflammation or degenerated adult helminth can be found (6).

Treatment includes use of antihelminthics such as mebendazole or albendazole (7).

## Conclusion

*E. vermicularis* is very common parasitic infestation, yet ectopic locations of its presence are uncommon. Physicians consider this parasitosis as possible cause of common symptoms such as abdominal and pelvic pain in order to provide adequate medical management including avoiding unnecessary surgical procedures, differentiation from malignancies and treatment of parasitosis in time in order to avoid further complications. It is important to recognize infestation of female genital tract to prevent serious complications and preserve female reproductive health.
